# Sensitivity and Specificity of Different Prognostic Systems in Guiding Surveillance for Metastases in Uveal Melanoma

**DOI:** 10.3390/cancers15092610

**Published:** 2023-05-04

**Authors:** Helena Robinson, Antonio Eleuteri, Joseph J. Sacco, Rumana Hussain, Heinrich Heimann, Azzam F. G. Taktak, Bertil Damato, Alexander J. Thompson, Thomas Allen, Helen Kalirai, Sarah E. Coupland

**Affiliations:** 1 Department of Clinical Engineering, Liverpool University Hospitals NHS Foundation Trust, Liverpool L7 8YE, UK; helena.robinson2@nhs.net; 2 NHS Digital, Liverpool University Hospitals NHS Foundation Trust, Liverpool L7 8YE, UK; 3 Liverpool Ocular Oncology Research Group, Department of Molecular and Cancer Medicine, University of Liverpool, Liverpool L7 8TX, UK; 4 Liverpool Ocular Oncology Centre, Liverpool University Hospitals NHS Foundation Trust, Liverpool L7 8TX, UK; 5 Department of Clinical Engineering, University Hospitals Bristol and Weston NHS Foundation Trust, Bristol BS2 8HW, UK; 6 Consultant Ocular Oncologist, St Erik’s Eye Hospital & Karolinska Institutet, 171 64 Stockholm, Sweden; 7 Manchester Centre for Health Economics, Division of Population Health, Health Services Research and Primary Care, The University of Manchester, Manchester M13 9PL, UK

**Keywords:** uveal melanoma, prognosis, liver surveillance, risk stratification

## Abstract

**Simple Summary:**

Uveal melanoma (UM) is an eye cancer that will spread to other parts of the body in almost 50% of cases, most commonly to the liver. Regular liver scans can lead to early detection of UM metastases. Current guidelines recommend such liver surveillance in UM patients with a ‘high risk’ of metastasis but do not specify how this group is defined. Several different systems can estimate a patient’s risk of dying from metastatic UM. Our study compared the accuracy of different UM prognostic systems when used to target enrolment into surveillance programmes and suggests that some systems could relieve some patients from unnecessary scans and conserve resources. We found that using the Liverpool Uveal Melanoma Prognosticator Online III (LUMPOIII) could offer equal sensitivity and greater specificity than other systems. We suggest guidance for its use, even when genetic testing is not provided. This study provides important context for revising the clinical guidelines for stratification for surveillance in UM.

**Abstract:**

Uveal melanoma (UM) metastasises in ~50% of patients, most frequently to the liver. Surveillance imaging can provide early detection of hepatic metastases; however, guidance regarding UM patient risk stratification for surveillance is unclear. This study compared sensitivity and specificity of four current prognostic systems, when used for risk stratification for surveillance, on patients treated at the Liverpool Ocular Oncology Centre (LOOC) between 2007–2016 (*n* = 1047). It found that the Liverpool Uveal Melanoma Prognosticator Online III (LUMPOIII) or Liverpool Parsimonious Model (LPM) offered greater specificity at equal levels of sensitivity than the American Joint Committee on Cancer (AJCC) system or monosomy 3 alone, and suggests guidance to achieve 95% sensitivity and 51% specificity (i.e., how to detect the same number of patients with metastases, while reducing the number of negative scans). For example, 180 scans could be safely avoided over 5 years in 200 patients using the most specific approach. LUMPOIII also offered high sensitivity and improved specificity over the AJCC in the absence of genetic information, making the result relevant to centres that do not perform genetic testing, or where such testing is inappropriate or fails. This study provides valuable information for clinical guidelines for risk stratification for surveillance in UM.

## 1. Introduction

Uveal melanoma (UM) is the most common primary intraocular cancer in adults and 600–800 people are diagnosed with this malignancy each year in the UK [1]. The primary tumour is normally treated with surgery, radiotherapy or a combination, almost always leading to local control [2]; however, it is metastatic spread, most commonly to the liver, that ultimately leads to the death of patients [1,3]. Several studies have demonstrated that surveillance consisting of regular non-ionising liver scans can effectively detect hepatic metastasis before the onset of symptoms [4,5,6].

Metastatic UM is associated with a poor prognosis; however, there are now several different licensed treatments that may prolong life (see [7] for a comprehensive review). These include liver-directed approaches such as surgical removal or ablation of metastases, and hepatic arterial infusion therapy [8,9,10,11,12,13] There is also an increasing number of systemic therapies available; phase II trials of nivolumab and ipilimumab used in combination have showed 12-month overall survival (OS) of >50% [14,15]. A recent randomised phase III trial of tebentafusp demonstrated a median OS duration of 21.7 months versus 16 months (*p* < 0.001) with investigators choice of therapy [16,17]. These developments mean that earlier detection of UM metastases provides patients with more therapy options than before, and enables patients to be enrolled in further clinical trials [1].

**Figure 1:**
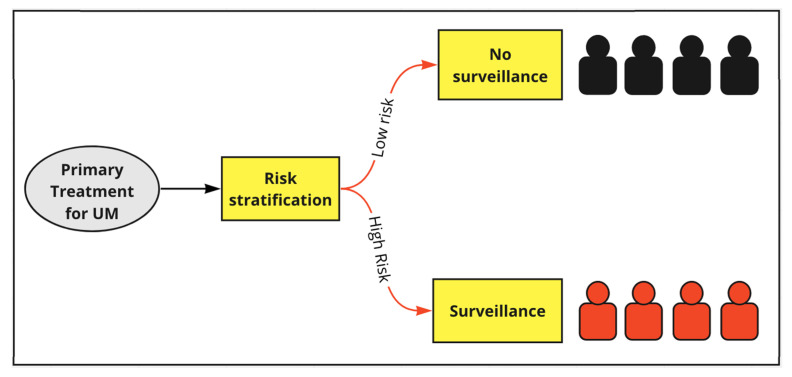
Diagram to illustrate the principle of risk stratification following primary treatment. The goal of stratification for surveillance is that all the patients who develop metastatic UM (red) are entered into the surveillance program, and all those who do not develop metastatic UM (black) are not. This is irrespective of the specific surveillance regime used.

The current UK guidelines for UM suggest that enrolment in surveillance is focused on patients with a ‘high risk’ of metastatic spread and that prognostication should be multifactorial, taking into account any clinical, histological and genetic features that are collected (Figure 1; [1]). However, the current guidance does not specify how the ‘high-risk’ cohort of UM patients is defined and there has been little comparative work to determine which systems should be used in UM risk stratification, or to suggest guidelines to facilitate adoption [1]. *This is the area of unmet need addressed by this study*. There are several prognostic systems available to clinicians and the systems compared by this study are described in Table 1. It is evident that over the last decade much progress has been made in identifying prognostic factors and developing systems and mathematical models to effectively synthesise this information [18].

The Ocular Oncology Biobank (OOB), University of Liverpool was established through close links with The Liverpool Ocular Oncology Centre (LOOC), Liverpool University Hospitals NHS Foundation Trust (LUHFT), one of England’s three specialist centres for UM treatment. It includes many patients, all of whom have consented for clinical, histological, genetic and survival data to be collected, prospectively. Here, we used this dataset to compare the performance of using monosomy 3 alone with clinical staging, and two approaches incorporating both monosomy 3 and clinicopathologic features (Table 1). The primary aim of this study was to use the LOOC data to provide sensitivity and specificity estimates for the different UM prognostic systems, when specific thresholds are used for stratification, to provide a resource for the development of surveillance guidelines. Secondary aims included repeating this analysis with all genetic data removed from the dataset to provide results relevant to centres that do not routinely perform genetic testing (or where this is not available for other reasons), and to provide an additional validation for all the prognostic systems considered.

## 2. Materials and Methods

### 2.1. Dataset

The Ocular Oncology Biobank (OOB) dataset, which contains all consented choroidal melanoma patients treated at Liverpool Ocular Oncology Centre (LOOC) was used for this study. The data were extracted for analysis on 23 May 2022.

Patients were included in the study only if they received a standard primary treatment (enucleation, plaque radiotherapy, local resection or endoresection with plaque radiotherapy, proton beam radiotherapy or photodynamic therapy) between 2007–2016. Patients were only included if they had at least 5 years of follow up, or when death from, or detection of metastasis was observed within this time; moreover, patients were excluded if they had missing data in any of the age, sex, tumour dimensions, ciliary body involvement or extraocular extension fields. Patients with iris melanomas were excluded, as iris melanomas have disease characteristics that are distinct from choroidal melanomas and have differing prognoses [4]. This dataset is largely independent of that used to construct the LUMPO III model (up to 398 patients may be present in both datasets, Figure S2).

Primary tumour location and size were determined at LOOC by ophthalmoscopy and slit-lamp examination. Tumour dimensions were measured by ultrasonography (US), which was also used to detect any extraocular spread. Routine histological analysis determined the presence or absence of epithelioid cells and of PAS+ closed connective tissue loops as well as mitotic count per 40 HPF. Chromosomal results were determined by Multiplex Ligation-dependent Probe Amplification (MLPA; *n* = 582), with a small number of results being determined by Microsatellite Analysis (MSA; *n* = 136) and some earlier cases by Fluorescence In Situ Hybridisation (FISH; *n* = 4 [30,31]). Outcome data (death, cause of death, detection of metastasis or date of the last follow up) were obtained from the National Cancer Registry and local hospital databases.

The study was reviewed by a Research Ethics Committee (REC [15/SC/0611]) and approved by the Health Research Authority (HRA) as an amendment to an existing approved protocol, ‘Enhancing prognostication in uveal melanoma’ (IRAS ID 187211). The OOB falls under the University of Liverpool HTA license (12020) and has HRA approval for collecting and storing patient data for research (REC 21/NW/0139). The study design is retrospective and observational and did not change the surveillance or treatment received by patients.

### 2.2. Distribution of Risk Scores and Classifications in the Patient Population

The four different prognostic systems compared with respect to stratification for surveillance in this study were: LUMPOIII, LPM, the AJCC staging system and monosomy 3 as a single marker. They are described in Table 1 together with their respective inputs and outputs. The 5-year metastasis-associated mortality (MAM) prediction outputs from LUMPOIII and LPM were calculated for each patient in the dataset. Five-year MAM has been used previously in studies regarding stratification for surveillance [5,8]. Similarly, each patient’s tumour was staged using the AJCC system following its classification rules [24]. The monosomy 3 system tested in this study, stratifies both those cases where monosomy 3 is present and those cases which lack a chromosome 3 result as requiring surveillance (Figure S1). The distribution of scores and classifications in the population were visualised using histograms and bar charts. Calculations and visualization were conducted using R (version 4.1.2). All coding for data preparation and analysis is available on GitHub (https://github.com/helenajr/um_stratificaton) (accessed on 28 April 2023).

### 2.3. Sensitivity and Specificity of the Four Prognostic Systems for Risk Stratification at LOOC

Stratification of the population for surveillance using each prognostic system, was simulated by setting thresholds, which divided those below the threshold into a low-risk (no surveillance) category and those above the threshold into a high-risk (surveillance) category. The principle of stratification is illustrated in Figure 1. The defined endpoint for all sensitivity and specificity analysis in this study was ‘death from; or detection of UM metastasis within 5 years of primary treatment’. It should be noted that LUMPOIII was designed to predict the endpoint of death from metastasis only, whereas, here, the detection of metastasis is included in the endpoint to maximise the clinical relevance of findings for a surveillance context [20]. For each system and threshold considered, patients correctly classified as high risk (i.e., they were above the threshold and the endpoint was observed) were considered true positives. Likewise, patients correctly classified as low risk (i.e., they were below the threshold and the endpoint was not observed) were considered ‘true negatives’. Patients incorrectly classified as high-risk were considered ‘false positives’. Patients incorrectly classified as ‘low risk’ were considered ‘false negatives’.

Sensitivity and specificity were calculated using both point estimates and receiver operating characteristic curve (ROC) analyses. ROC analysis was used only for the prognostic systems which gave a continuous risk score (LUMPOIII and LPM), as it enables the visualization of all possible thresholds. The formulae used for point estimates were as follows:

Sensitivity = True Positives/(True Positives + False Negatives)(1)

Specificity = True Negatives/(True Negatives + False Positives)(2)

Confidence intervals (CIs) for point estimates of sensitivity and specificity were calculated using the Clopper-Pearson exact CI method from the PropCIs package (version 0.3.0; [32]). ROC analysis was conducted and visualised using the R package plotROC (version 2.3.0; [33]). Area Under the Curve (AUC) and 95% Cis were calculated using the same package. Interpretation of the Area Under the Curve (AUC) resulting from the ROC analysis was 1 indicated perfect discrimination, 0.9–0.99 was excellent discrimination, 0.8–0.89 was good, 0.7–0.79 was fair and discrimination < 0.7 was considered poor [34]. ROC curves and point estimates were visualized within the same plot to facilitate comparison between systems and thresholds. AUCs were compared using DeLong paired test, implemented in the roc.test function from the package pROC (version 1.18.0). All *p*-values were adjusted using the Holm correction for multiple testing to control the family-wise error rate, using the p.adjust function.

#### 2.3.1. Subgroup Analysis of LOOC Patients with and without a Chromosome 3 Result

The dataset was divided into two subgroups. Those patients with known chromosome 3 status (irrespective of status) and patients with unknown chromosome 3 status. The subgroups were assessed for differences in the incidence of the endpoint and differences in tumour size. For the prognostic systems that use chromosome 3 information as part of their input (LUMPOIII, LPM), the sensitivity and specificity analyses described in Section 2.3 were then conducted separately on each subgroup to allow for selection of a threshold offering high sensitivity for each subgroup.

#### 2.3.2. Decision Algorithm for Implementing Strategies Incorporating Two Thresholds

The selected thresholds from the subgroup analysis for LUMPO and LPM were combined into simple decision algorithms, illustrated using a flowchart, to illustrate how the thresholds could be applied to the whole population [35]. The dataset (including both subgroups) was then stratified using the simple algorithm defined in the flowchart. The overall sensitivity and specificity of this strategy was then analysed, using the methodology described in Section 2.3 and the results compared to other strategies.

#### 2.3.3. Comparison of Health Economic Impact of Using Different Strategies

The real-world impact of using different strategies was compared in terms of numbers of patients undergoing unnecessary surveillance (false positives, as defined in Section 2.3), numbers of patients incorrectly stratified as low risk (false negatives, as defined in Section 2.3), numbers of scans required and monetary cost of those scans.

All results in this section are based on a population of 200 UM patients (the approximate number seen by LOOC in a year), an incidence of the endpoint of 28% (derived from the dataset) and a surveillance programme of 6-monthly non-contrast MRI scans. This reflects current practice at LOOC and the efficacy of the surveillance programme has been investigated in previous studies [5,8]. The number of false negatives and false positives were calculated as follows and figures rounded to the nearest whole number:

Total positives = Population size * Incidence of endpoint

Total negatives = Population size * (1-Incidence of endpoint)

True positives = Total positives * Sensitivity

False negatives = Total positives * (1-Sensitvity)

True negatives = Total negatives * Specificity

False positives = Total negatives * (1-Specificity)

The calculation of the number of scans delivered under each strategy for a population of 200 patients over 5 years made the following assumptions: true and false negatives were assumed to receive no scans; false positives were assumed to receive 10 scans over 5 years; and true positives were assumed to receive 3 scans. In calculations of monetary cost of delivering these scans, the cost of one episode of surveillance was taken from the 2020/21 NHS England National Schedule of Costs. The figures used were for a magnetic resonance image (MRI) of one area without contrast in an outpatient aged 19 years or over (£211.24). Costs for an outpatient ultrasound (US) scan without contrast, with a duration of 20 min or more (£135.09) are also used, as this is a modality favored by many centres [36].

### 2.4. Sensitivity and Specificity of Prognostic Systems for Risk Stratification at a Centre That Does Not Offer Genetic Testing

Several ocular oncology centres lack the facility for routine genetic testing of primary UM. Therefore, it was important to additionally compare the risk stratification methods where the whole population was lacking a chromosome 3 result, rather than just a subset of the population. To simulate data for a centre that does not routinely offer genetic testing (i.e., none of the patients will have known chromosome 3 or chromosome 8q status), the data in these fields were removed from all patients in the dataset. LUMPOIII and LPM (which use these fields as input) risk scores were then re-calculated with this information lacking.

The sensitivity and specificity comparison of LUMPOIII and LPM versus AJCC in this context were then conducted using the same methodology as described in Section 2.3 and a comparison of the health economic impact was carried out using the same methodology as described in Section 2.3.3.

## 3. Results

### 3.1. Description of the Dataset Characteristics

After exclusion criteria had been applied (see Methods) a total of 1047 patients were included in the dataset (Table 2). Descriptive statistics showed a median age of 61 and that 53% of the patients were male. 292 patients in the dataset died from UM metastases and/or metastatic spread was detected within 5 years. For those experiencing either of these endpoints, the median time to endpoint in the dataset was 19 months. Table 2 shows that enucleation and plaque radiotherapy were the most common primary treatments in this study period at LOOC.

At LOOC, UM samples are routinely tested (after patient consent) to determine chromosome 3 status; such analysis was recorded in most patients in this dataset (*n* = 723). Around a third of patients (*n* = 324) in the dataset had no chromosome 3 result recorded. This includes patients with either small tumour size, such that there was insufficient DNA for chromosomal analysis, as well as patients declining intraocular biopsy. Hence, in the dataset, this information is not missing at random and there are some differences in characteristics of the subgroups of patients at LOOC with and without a chromosome 3 result. The subgroup of patients lacking a chromosome 3 result had smaller tumours on average, and a lower incidence of death from metastasis or detection of metastasis within 5 years of primary treatment, (14% versus 34%, respectively, Figure S3).

### 3.2. Distribution of Risk Scores and Classifications in the Patient Population

**Figure 2:**
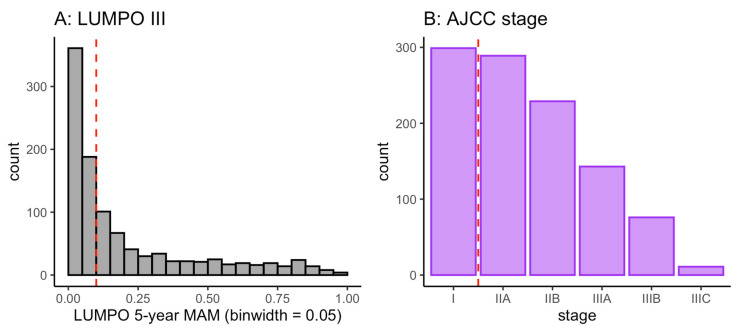
Distribution of scores or risk categories of LOOC patients in the dataset. (**A**) Histogram showing distribution of LUMPO III 5-year MAM (metastatic associated mortality) predictions and an example threshold of 0.1 (dashed red line). (**B**) Bar chart showing distribution of AJCC stages in the dataset and an example stratification threshold of stage ≥ IIA.

As detailed in the Methods section, each patient in the dataset was scored or classified under each of the four systems (LUMPOIII, LPM, the AJCC system and monosomy 3). Plotting the distribution of the resulting scores or classifications showed that the patient population treated at LOOC was skewed towards lower risk scores or categories (Figure 2). The skewed distribution means that the stratification threshold used can be relatively low, and still be effective in classifying many patients as low risk.

### 3.3. Sensitivity and Specificity of the Four Prognostic Systems for Risk Stratification at LOOC

**Figure 3:**
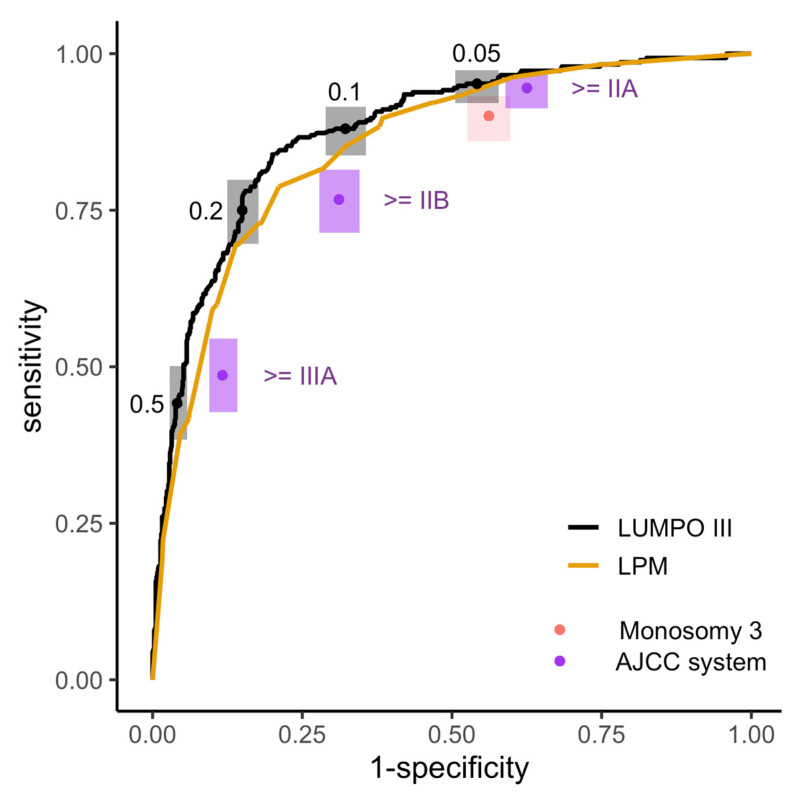
Comparison of discrimination performance of 4 systems of risk stratification for surveillance on the LOOC patient population. Empirical ROC analysis of the 5-year MAM score provided by LUMPOIII (black line; AUC = 0.88 [0.85–0.9]) and LPM (yellow line; AUC = 0.85 [0.82–0.88]). Point estimates (points) are shown with 95% CIs (boxes) of sensitivity and specificity for using the monosomy 3 system (pink), the AJCC system with thresholds (purple, labelled), or LUMPOIII with thresholds (black, labelled).

The sensitivity and specificity results for the four prognostic systems are displayed in Figure 3 and Table 3. LUMPOIII and LPM give risk scores that were continuous and therefore have many possible thresholds, and the ROC curves show the sensitivity and specificity at all possible thresholds. The ROC analyses demonstrated that both LUMPOIII (Area under the curve [AUC] = 0.88 [0.85–0.9]) and LPM (AUC = 0.85 [0.82–0.88]) showed good discrimination ability on this dataset. The difference between the AUC of LUMPOIII and LPM had an adjusted *p*-value of 0.003.

Table 3 provides a summary of the sensitivity and specificity of the different systems at certain thresholds. Raising the threshold increased the specificity at the expense of sensitivity, and vice versa. Comparing these systems showed that using LUMPOIII with a threshold of ≥0.05 (i.e., where a patient with a score higher than 0.05 is enrolled in the surveillance programme), achieved better specificity, for the same high level of sensitivity than could be achieved with the AJCC classification system (Figure 3, Table 3). Likewise, using LUMPOIII with a threshold of ≥ 0.1 achieved greatly increased specificity, for the same level of sensitivity than could be achieved using the monosomy 3 system (Figure 3, Table 3). In practice, this means that by switching from using AJCC or monosomy 3 to using LUMPOIII with the thresholds described above, the same number of cases can be detected with fewer people enrolled in surveillance. This analysis used the same threshold for all the patients in the dataset; however, the following section considers whether different thresholds are appropriate for patients with and without a chromosome 3 result.

#### 3.3.1. Subgroup Analysis of LOOC Patients with and without a Chromosome 3 Result

Where it is in accordance with patient consent, LOOC routinely tests for monosomy 3, which is known to have strong prognostic value and thus have a strong effect on the MAM predictions provided by the models [20]. Therefore, when this information is missing the MAM predictions produced by LUMPO III or LPM are less accurate. As detailed in Section 3.1, a non-random subpopulation of LOOC patients has no chromosome 3 result. Using the same threshold for both subpopulations, for example LUMPOIII MAM ≥ 0.05, would result in lower sensitivity and specificity for the subpopulation of patients without a chromosome 3 result (Table S1).

**Figure 4:**
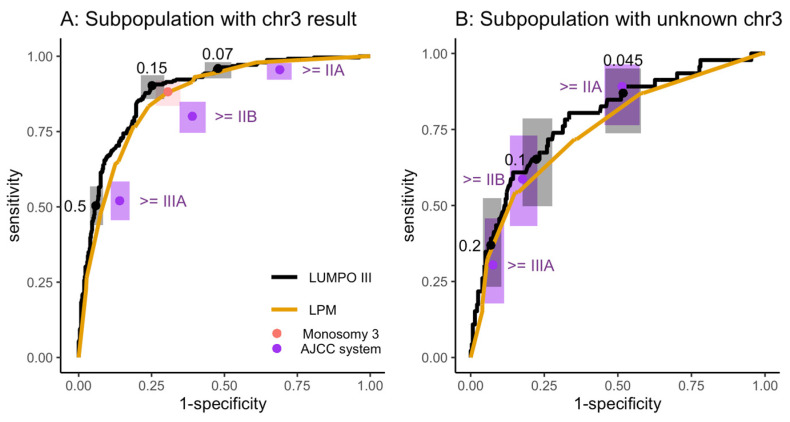
Comparison of discrimination performance of the 4 systems of risk stratification for surveillance on the LOOC patient population. Empirical ROC analysis of the 5-year metastatic associated mortality score provided by LUMPOIII (black line) and LPM (yellow line). Point estimates are shown with 95% CIs (points with boxes) of sensitivity and specificity for using the monosomy 3 system (pink), the AJCC system with thresholds (purple, labelled), or LUMPOIII with thresholds (black, labelled). (**A**) Subpopulation with a chromosome 3 (chr3) result. (**B**) Subpopulation lacking chromosome 3 results.

Therefore, it was deemed important to conduct a subgroup analysis, with the expectation that a different threshold may be appropriate for the two subgroups. As expected, LUMPOIII (AUC = 0.88 [0.86–0.91]) and LPM (AUC = 0.86 [0.83–0.89]; adjusted *p*-value = 0.007) showed much better discrimination in the subpopulation with a chromosome 3 result, than the subpopulation without (AUC = 0.79 [0.71–0.87], AUC = 0.75 [0.66–0.83], respectively; adjusted *p*-value = 0.06, Figure 4).

In the subpopulation with a chromosome 3 result, stratifying using LUMPOIII with a threshold of 5-year MAM ≥ 0.07 showed equal sensitivity and greatly increased specificity (52%) than stratifying using AJCC stage of ≥IIA (31% specificity, Figure 4A). In the subpopulation without a chromosome 3 result, using a more conservative threshold of LUMPOIII 5-year MAM ≥ 0.045, showed equivalent sensitivity and specificity to stratifying using AJCC stage of ≥IIA (Figure 4B).

#### 3.3.2. Decision Algorithm for Implementing Strategies Incorporating Two Thresholds

**Figure 5:**
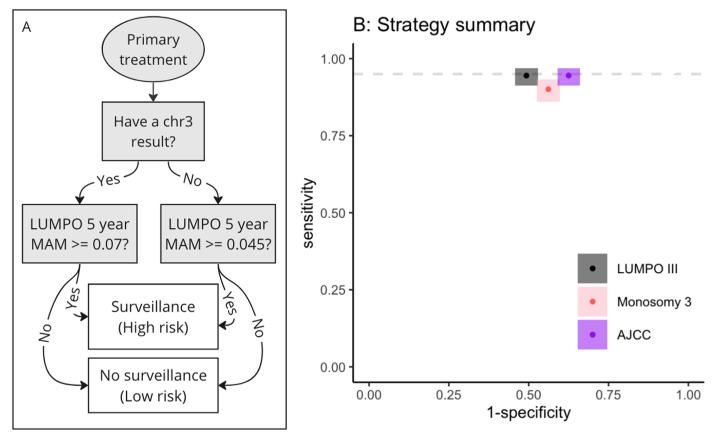
Flowchart showing a simple decision algorithm for using LUMPOIII with a different threshold for those patients lacking a chr3 result and comparison with other systems. (**A**) Flowchart demonstrating how to use LUMPOIII with a different threshold for those with and without chromosome 3 information. (**B**) Point estimates of sensitivity and specificity using this strategy (black), compared with stratifying using the monosomy 3 system (pink) and AJCC stage ≥ IIA (purple). Grey dashed line shows 95% sensitivity.

In practice two thresholds can be used in a simple decision algorithm, where the threshold used depends on the availability of chromosome 3 status information. This was illustrated using a simple flowchart (Figure 5A). When compared to the AJCC or monosomy 3 systems, using this strategy had equal sensitivity, but greater specificity than risk stratification using an AJCC stage of ≥IIA, and it had high sensitivity for all patients irrespective of the availability of chromosome 3 information (Figure 5, Table 4). It also had better sensitivity and specificity than using chromosome 3 status alone and better specificity than using a single threshold for all patients. Table 4 summarises this information and compares the performance of several possible strategies using LUMPOIII or LPM with different threshold values depending on the availability of the chromosome 3 result.

#### 3.3.3. Comparison of Health Economic Impact of Using Different Strategies

Given a population of 200 UM patients, using the LUMPOIII strategy shown in Figure 5 and Table 4 (No. 1), instead of AJCC stage, would relieve 18 patients from unnecessary surveillance (i.e., 18 fewer false positives, Table 5), equating to 180 fewer scans over a 5-year period. Assuming a cost of £211.24 for a non-contrast MRI of the liver over a 5-year period, this equals a cost saving of £2112.4 per patient and £38,023 overall [36]. If ultrasound was used as the imaging modality, at a cost of £135.09 per scan, the cost saving would be £24,316 over 5 years. Given equal sensitivity, an equal number of cases would be detected and treated (equal numbers of true positives and false negatives, Table 5).

Similarly, using strategy No. 2 shown in Table 4 (thresholds of LUMPOIII 5-year MAM ≥ 0.15 and ≥0.045 for those with and without a chromosome 3 result, respectively) compares favourably with using the monosomy 3 system. In the population of 200 patients, using this LUMPOIII strategy would relieve 31 patients from unnecessary surveillance, equating to 310 scans over a 5-year period and a cost saving of £65,484 (MRI) or £41,877 (US), when compared with using chromosome 3 status alone.

### 3.4. Sensitivity and Specificity of Prognostic Systems for Risk Stratification at a Centre which Does Not Offer Genetic Testing

**Figure 6:**
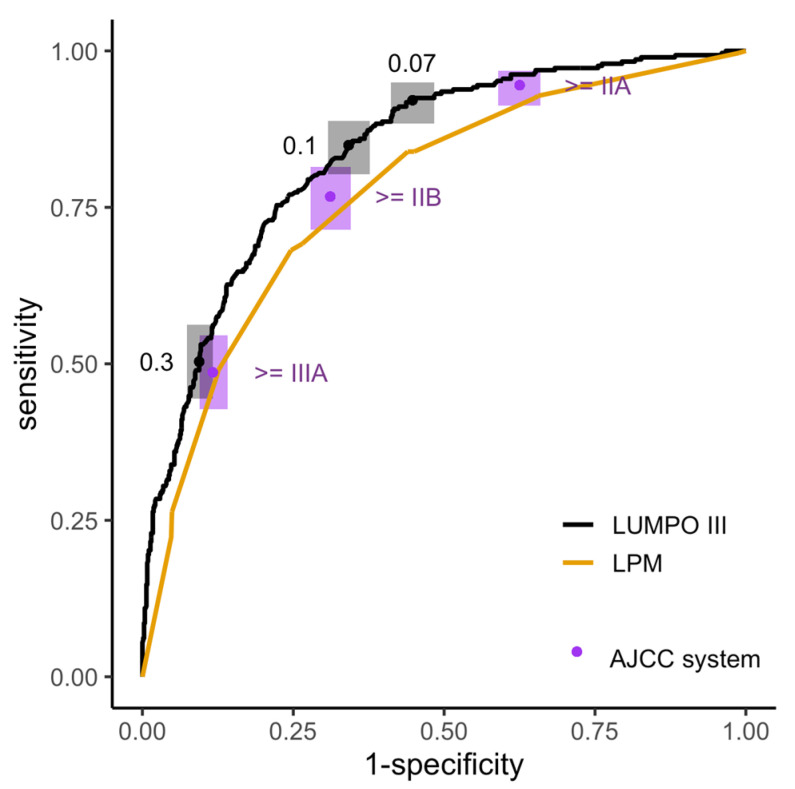
Comparison of discrimination performance of the 3 systems of risk stratification for a centre without a genetics service. Empirical ROC analysis of the 5-year metastatic associated mortality score provided by LUMPOIII (black line; AUC = 0.84 [0.81–0.87]) and LPM (yellow line; AUC = 0.77 [0.74–0.81]). Point estimates (points) are shown with 95% CIs (boxes) for using the AJCC system with thresholds (purple, labelled), or LUMPOIII with thresholds (black, labelled).

As expected, LUMPOIII discrimination performance was decreased in the absence of any genetic inputs, but it was still considered good (AUC = 0.84 [0.81–0.87]; Figure 6, Table 6). LPM performance was further decreased and only considered fair in this context (AUC = 0.77 [0.74–0.81]; adjusted *p*-value for the comparison ≤ 0.0001).

As was the case in the previous section, it was possible to employ different thresholds using the LUMPOIII and AJCC systems to achieve different levels of sensitivity and specificity (Table 6). As an example, using LUMPOIII with a threshold of ≥0.07 to enrol patients in surveillance showed much improved specificity than classifying using a AJCC stage of ≥IIA (Figure 6). In terms of the impact analysis this means that in a population of 200 patients, using this LUMPO III strategy would relieve 26 patients from unnecessary surveillance, equating to 260 scans over a 5-year period and a cost saving of £54,922 (MRI) or £35,123 (US). As before, given equal sensitivity of these approaches would mean an equal number of cases are detected and treated.

## 4. Discussion

This study demonstrates how choice of risk stratification method could relieve patients from unnecessary liver surveillance and allow more effective use of available resources. Our study found that stratification using LUMPOIII or LPM offered equal sensitivity and greater specificity than stratification with the AJCC system or the monosomy 3 system. It also details the threshold values for LUMPOIII or LPM that should be used to achieve higher specificity without loss of sensitivity, and quantified the patient and cost benefits of this. Additionally, this study found that LUMPOIII could offer greater specificity at high levels of sensitivity in the absence of any genetic testing, which is relevant for centres that do not routinely carry out these tests. To our knowledge, this is the first study to compare the performance of available prognostic systems on the same dataset, focused on use for risk stratification. This analysis provides important context for clinicians using LUMPOIII for risk stratification, and more widely for revising future UM surveillance guidelines.

This study has several strengths. The dataset used in the analysis was large, collected prospectively over 10 years and was of high data quality, so it is likely to be highly representative of ‘real world’ UM patient populations. This study addresses an unmet need regarding how best to use available prognostic tools for risk stratification and puts forward suggestions as to how these tools could be implemented with >90% sensitivity, focusing this study on practical questions of concern to clinicians and patients. While strategies for using LUMPO III were suggested by this study, we understand there may be reasons for using a different prognostic system or different thresholds. This study therefore provides a high level of detail about the performance of each system at several different thresholds to provide a comprehensive reference for clinicians and patients interested in this question.

It is well known in the ocular oncology field that surveillance strategies vary across centres in the UK and the globe, despite attempts to try to make them more uniform using national or international guidelines. One reason for this is the persisting debate as to what defines a metastatic high-risk UM patient. At present, therefore, it is not known what proportion of UM patients receive regular surveillance, how often, and by what modality (or modalities). Our study proposes that high-risk UM patients would be best-defined using a multiparametric algorithm, such as LUMPOIII, and that in accordance with surveillance in other cancers, there are thresholds to aid surveillance strategies, as proposed in Figure 5A and Table 6. That is, if surveillance of 200 patients with routine testing of chromosome 3 status was undertaken using the scenario of Figure 5A, then three patients with metastatic UM would be missed (false negatives). Likewise, in a scenario of no routine chromosome 3 testing, using the lowest threshold from Table 6, three patients would be missed. Both approaches have the same sensitivity, and hence the same number of false negatives (missed cases). The Liverpool approach (with regular testing of chr3) has better specificity, and therefore fewer false positives (unnecessary surveillance). Whether these numbers are acceptable to the ocular oncology field and their patients requires further discussion.

We acknowledge that there are some limitations to this study as well as areas for future work. This study used a 5-year time horizon for the analysis endpoint (i.e., death from, or detection of metastasis). Although most patients developing UM metastases do so during this time period, it is well known that a proportion of patients will develop detectable metastases more than 5 years after treatment [37,38]. Therefore, repetition of this analysis using longer follow-up periods would be beneficial when more follow-up data becomes available. This limitation is also important to consider when using this analysis to inform clinical decisions, particularly when considering younger UM patients.

The LUMPOIII and LPM models were developed to predict the endpoint of death from metastatic UM and not detection of metastasis. This endpoint is different but closely related to the endpoint used in this study (death from UM spread, or detection of metastasis), which was chosen to maximise clinical relevance for use in stratification for surveillance. Although this study showed that the models still performed very well at predicting this related outcome, future models, specifically trained on the endpoint most relevant to stratification for surveillance could be developed. A recent study advanced work in this area, making a novel model using some predictors from the LUMPOIII model as inputs to predict the onset of detectable metastatic disease [39]. Furthermore, LUMPOIII’s parameters do not incorporate the most up-to-date genomic alterations of UM cells: i.e., mutations in *BAP1*, *SF3B1*, and *EIF1AX*) [40], which affect clinical outcome. However, the LUMPO algorithm does allow for iterative improvements as soon as sufficient data can be incorporated into it. Such a modified LUMPO could be of value for patient stratification and clinical trial entry.

Finally, our study was limited to comparing the relative sensitivity and specificity of different prognostic systems, rather than suggesting an optimum level of sensitivity and specificity. This could be addressed in future by a full economic analysis of different strategies, which requires accurate costs and benefits to be calculated for all elements of the surveillance and subsequent diagnostic and treatment pathways. This is currently challenging because there are several different treatment options for UM metastases and patients may undergo multiple types of therapy. Another problem is that many of the treatment options are supported by small studies, and have confidential pricing agreements for use in the NHS [8,11,41]. In addition, many patients are enrolled in clinical trials, for which the costs and benefits are difficult to quantify. There is also a lack of data surrounding the psychological cost or benefit of being enrolled in liver surveillance, as well as patient compliance with the surveillance programmes, which can all have a significant impact on economic evaluations [42]. Considering such uncertainty, the study team decided that high levels of sensitivity, detecting ≥90% of cases was desirable. The suggested strategies detailed in this paper are consistent with this objective.

## 5. Conclusions

This study demonstrated how changing risk stratification method could increase specificity without impacting sensitivity, thereby detecting the same number of cases with fewer scans. It found that using LUMPOIII or LPM provided superior performance in this regard than using the AJCC system or a monosomy 3 system and suggested guidance for using LUMPOIII to achieve 95% sensitivity and 51% specificity. Even for centres that do not offer genetic testing, this study found that LUMPOIII could still offer greater specificity at the high levels of sensitivity than the current AJCC system. This study provides important context for deciding and improving upon current risk stratification strategies; however, further work is needed to better understand the benefits and costs of surveillance for patients to determine an optimum strategy.

## Figures and Tables

**Table 1:** Systems for prognostication of uveal melanoma compared in this study.

System	Description	Inputs	Outputs
**Liverpool Uveal Melanoma Prognosticator Online (LUMPOIII)**	LUMPO III is a semiparametric Markov multi-state model developed using a large dataset of UK patients [19,20]. It has been externally validated on datasets from different centres and is available to clinicians via a website [21,22].	Age, sex, tumour diameter, tumour height, ciliary body involvement, extraocular extension, presence of epithelioid cells, presence of closed Periodic Acid Schiff (PAS)-positive connective tissue loops, mitotic count (per 40 high power field [HPF]), monosomy 3, chromosome 8q gain	Probability (0–1) of death from metastasis (metastatic associated mortality [MAM]) and probability of death from other causes for each year up to 10 years after primary treatment
**Liverpool Parsimonious Model (LPM)**	LPM was developed from the same dataset as the LUMPOIII model [23]. Due to its relative simplicity, prognostication can be conducted using just a reference table, improving accessibility.	Age, tumour diameter, monosomy 3	Probability (0–100) of death from metastasis for 2, 5 and 10 years after primary treatment
**The American Joint Committee on Cancer (AJCC) staging system; 8th Edition**	The AJCC system provides a universal staging system which has been adapted for use for cancer at any anatomical site [24,25].	Tumour diameter, tumour height, ciliary body involvement, extraocular extension	Seven ordinal primary tumour stages (I, IIA, IIB, IIIA, IIIB, IIIC)
**Monosomy 3 only system (Figure S1)**	Monosomy 3 (and underlying loss of function of the tumour suppressor gene BAP1) is a strong independent prognostic factor present in ~50% of choroidal melanomas [26,27,28,29].	Monosomy 3	Patients with monosomy 3 classified as ‘high risk’; patients with disomy 3 (normal) status classified as ‘low risk’; patients without a chromosome 3 result classified as ‘unknown’ risk (and also recommended surveillance)

**Table 2:** Descriptive statistics of dataset. All tumour characteristics relate to the primary UM. The endpoint definition for analyses is ‘death from metastasis or detection of metastasis within 5 years of primary treatment’.

Variable	Count	Median	Range	Number Missing
**Endpoint**	Endpoint: 292			
	No endpoint: 755			
**Age**	1047	61	18–94	-
**Sex**	F: 490	-	-	-
	M: 557			
**Largest tumour diameter (mm)**	1047	12.7	1.2–26	-
**Tumour height (mm)**	1047	4.5	0.5–18.3	-
**Ciliary body involvement**	Present: 210	-	-	-
	Absent: 837			
**Extraocular extension**	Present: 54	-	-	-
	Absent: 993			
**Epithelioid cell type**	Present: 463	-	-	158
	Absent: 426			
**Presence of PAS+ closed loops**	Present: 239	-	-	610
	Absent: 198			
**Mitotic count/40 HPF**	0–1: 53	-	-	602
	2–3: 143			
	4–7: 148			
	7+: 101			
**Chromosome 3 loss**	Present: 363	-	-	324
	Absent: 360			
**Chromosome 8q gain**	Present: 300	-	-	480
	Absent: 267			
**Primary treatment**	Enucleation: 371	-	-	-
	Plaque radiotherapy (RT): 343			
	Proton Beam RT: 231			
	Endoresection + Plaque RT: 49			
	Local resection + Plaque RT: 46			
	Photodynamic therapy: 7			

**Table 3:** Summary table of different risk stratification thresholds. Displays positive predictive value (PPV), negative predictive value (NPV) and the percentage of the total population defined as high-risk, and thus enrolled on surveillance (Surveillance), dependent on risk stratification system and threshold (5-year metastatic associated mortality is abbreviated to MAM).

System	Threshold	Sensitivity	Specificity	PPV	NPV	Surveillance
**LUMPOIII**	MAM ≥ 0.05	95% (92–97)	46% (42–49)	40%	96%	66%
**LUMPOIII**	MAM ≥ 0.1	88% (83–92)	68% (64–71)	51%	94%	48%
**LUMPOIII**	MAM ≥ 0.2	75% (79–82)	85% (82–87)	66%	90%	32%
**LPM**	MAM ≥ 5	93% (90–96)	48% (44–51)	41%	95%	64%
**LPM**	MAM ≥ 10	90% (86–93)	62% (58–65)	47%	94%	53%
**AJCC**	Stage ≥ IIA	95% (91–96)	37% (34–41)	37%	95%	71%
**AJCC**	Stage ≥ IIB	77% (71–81)	69% (65–72)	49%	88%	44%
**Monosomy 3**	NA	90% (86–93)	44% (40–47)	38%	92%	66%

**Table 4:** Summary table of strategies with different thresholds for patients with and without a chromosome 3 (Chr3) result. Three different strategies using LUMPOIII or LPM at different thresholds are shown. The table also displays positive predictive value (PPV), negative predictive value (NPV) and the percentage of the total population defined as high risk, and thus enrolled on surveillance (Surveillance), dependent on each risk stratification method and threshold.

No.	System	Threshold		Sensitivity	Specificity	PPV	NPV	Surveillance
		Known Chr3	Unknown Chr3					
1	LUMPOIII	MAM ≥ 0.07	MAM ≥ 0.045	95% (91–97)	51% (47–54)	43%	96%	62%
2	LUMPOIII	MAM ≥ 0.15	MAM ≥ 0.045	90% (86–93)	65% (62–68)	50%	94%	50%
3	LPM	MAM ≥ 11	MAM ≥ 7	92% (88–95)	54% (50–57)	43%	95%	59%
4	AJCC	Stage ≥ IIA	Stage ≥ IIA	95% (91–96)	37% (34–41)	37%	95%	71%
5	Monosomy 3	M3 or no result included in surveillance group		90% (86–93)	44% (40–47)	38%	92%	66%

**Table 5:** Reduction in false positives with LUMPO III (strategy No. 1; Table 4). Total numbers of true positives, false negatives, false positives and true negatives assuming a total population of 200 patients and an incidence of the endpoint of 28%.

System	Sensitivity	Specificity	True Positives	False Negatives	False Positives	True Negatives	Total
LUMPOIII	95%	51%	53	3	71	73	200
**AJCC**	95%	38%	53	3	89	55	200

**Table 6:** Summary table of different risk stratification methods for a centre without a genetics service. Displays positive predictive value (PPV), negative predictive value (NPV) and the percentage of the total population that are enrolled in surveillance (Surveillance).

System	Threshold	Sensitivity	Specificity	PPV	NPV	Surveillance
LUMPOIII	MAM ≥ 0.05	95% (91–97)	44% (40–47)	39%	95%	67%
LUMPOIII	MAM ≥ 0.07	92% (88–94)	55% (52–59)	44%	95%	58%
LUMPOIII	MAM ≥ 0.1	85% (80–89)	66% (62–69)	49%	92%	48%
LPM	MAM ≥ 5	93% (89–95)	34% (31–38)	35%	93%	73%
AJCC	Stage ≥ IIA	95% (91–97)	37% (34–41)	37%	95%	71%
**AJCC**	Stage ≥ IIB	77% (71–81)	69% (65–72)	49%	88%	44%

## Data Availability

This work uses data provided by patients and collected by the NHS as part of their care and support. The data used in this study are not publicly available, as they are special category personal data that carry a risk of re-identification. The corresponding author will consider reasonable requests for access. The analytic code used for analysis is publicly available at https://github.com/helenajr/um_stratificaton (accessed on 28 April 2023).

## Supplementary Materials

The following supporting information can be downloaded at: https://www.mdpi.com/article/10.3390/cancers15092610/s1, Figure S1: Flowchart illustrating the monosomy 3 system for risk stratification; Figure S2: Dataset in this study is largely independent from the dataset used to train LUMPOIII and LPM; Figure S3: chans between subpopulations with and without a chromosome 3 result; Table S1: Sensitivity and specificity estimates for each subpopulation when a threshold of LUMPOIII 5-year MAM ≥ 0.05 is used for stratification.
