# Supplementary material for: Sensitivity and Specificity of Different Prognostic Systems in Guiding Surveillance for Metastases in Uveal Melanoma
Source: Cancers (Basel). 2023 May 4;15(9):2610. doi: 10.3390/cancers15092610 (PMC10177440; doi:10.3390/cancers15092610)
Supplement: Supplementary file 1 [file cancers-15-02610-s001.zip › cancers-2322953-supplementary.pdf]

Supplementary materials

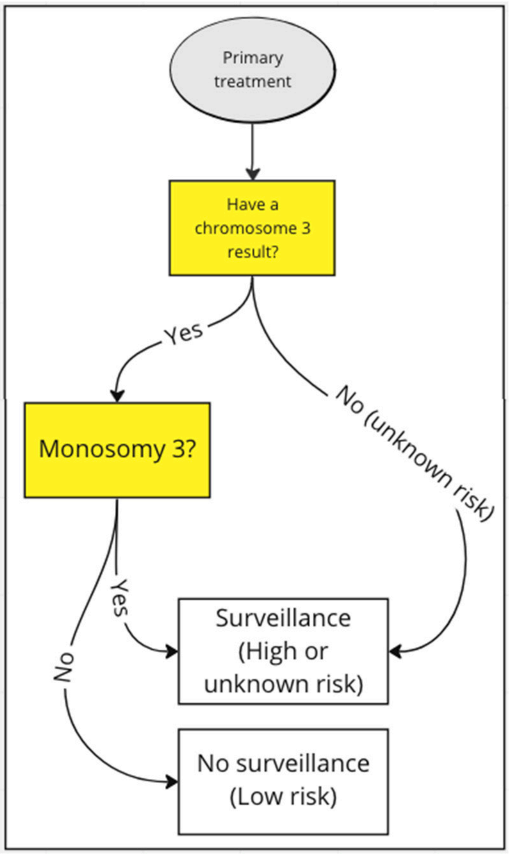

Figure S1. The 'monosomy 3' system analysed in this study, where both those patients with monosomy 3 present and those with no chromosome 3 result are enrolled in the surveillance programme.

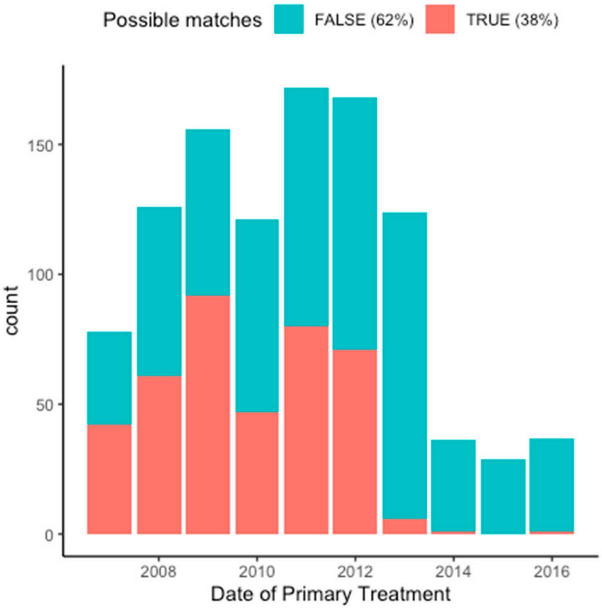

Figure S2. The dataset used to develop the LUMPOIII and LPM models was a different, anonymised dataset to that used in the present study, although it also contained patients treated at LOOC. As it is not possible to assess the overlap between datasets using identifiers, patients with the same sex, age at primary management, tumour basal diameter and height were identified as being possible matches. This chart shows the number of patients undergoing primary treatment in each year in the present study (blue), and the proportion of those which may also be present in the LUMPOIII model (red). It shows that the dataset used in this study is at least 62% independent of the dataset used to develop the model.

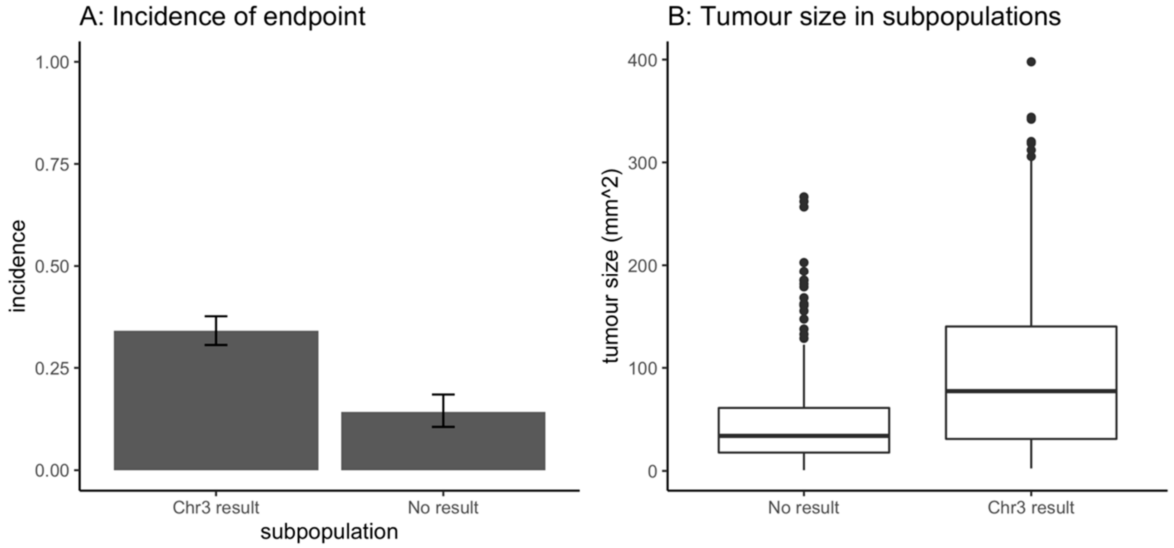

Figure S3. (**A**) Subpopulations with and without a chromosome 3 result have different prevalence of the outcome (death from or detection of metastasis within 5 years). (**B**) Subpopulations with and without a chromosome 3 result have differences in average size of tumour. Size was calculated as largest basal diameter * height

Table S1. Sensitivity and specificity estimates for each subpopulation when a cut-off of LUMPOIII 5-year MAM >= 0.05 is used for stratification.

Subpopulation	Sensitivity	Specificity
With chr3 result	97% (94-99)	42% (38-47)
Without chr3 result	85% (71-94)	51% (45-57)
